# Effect of type 2 diabetes on A disintegrin and metalloprotease 10

**DOI:** 10.1111/1753-0407.13287

**Published:** 2022-06-15

**Authors:** Sum Lam, Sammy Wing‐Ming Shiu, Ying Wong, Kathryn Choon‐Beng Tan

**Affiliations:** ^1^ Department of Medicine University of Hong Kong Hong Kong SAR China

**Keywords:** a disintegrin and metalloproteases, biomarker, lectin‐like oxidized‐LDL receptor, sheddase, 解整合素金属蛋白酶, 血凝素氧化低密度脂蛋白受体, 脱落酶, 生物标记物

## Abstract

**Background:**

As a type 1 transmembrane protein, a disintegrin and metalloprotease 10 (ADAM10) is responsible for the cleavage of a variety of cell surface molecules and has been implicated in the pathogenesis of Alzheimer disease, atherosclerosis, and inflammatory and neoplastic disorders. It has been suggested that systemic ADAM10 concentration may potentially be used as a prognostic biomarker. Since high glucose can upregulate ADAM10 expression in vitro, we investigated whether serum levels of ADAM10 and its substrate, the lectin‐like oxidized low‐density lipoprotein receptor 1 (LOX‐1), can be influenced by type 2 diabetes.

**Methods:**

A total of 1091 individuals with type 2 diabetes and 358 age‐matched healthy control subjects were recruited. Serum concentrations of ADAM10 and the soluble form of LOX‐1 (sLOX‐1) released by cleavage of LOX‐1 by ADAM were measured by enzyme‐linked immunosorbent assay kits (ELISA).

**Results:**

Serum ADAM10 was increased in subjects with diabetes compared with control (40.5 ng/mL [22.3‐65.7] vs 10.3 ng/mL [7.0‐17.9], respectively; *P* < .01); the highest levels were seen in insulin‐treated subjects. On multiple linear regression analysis, glycosylated hemoglobin, age, body mass index, and insulin use were independent determinants of ADAM10 level. The increase in serum ADAM10 levels in diabetes was accompanied by changes in serum sLOX‐1. Subjects with diabetes had higher serum sLOX‐1 than the control (110 pg/mL [89‐153] vs 104 pg/mL [85‐138], respectively; *P* < .01), and there was a significant correlation between serum ADAM10 and sLOX‐1 (*r* = 0.26, *P* < .01).

**Conclusions:**

Serum concentration of ADAM10 is increased in type 2 diabetes and is associated with glycemia and insulin therapy, which may potentially affect the specificity of systemic ADAM10 level as a biomarker.

## Graphical abstract

**Highlights**

- a disintegrin and metalloprotease 10 (ADAM10), a sheddase responsible for the cleavage of a variety of cell surface molecules, has been implicated in the pathogenesis of Alzheimer disease, atherosclerosis, and inflammatory and neoplastic disorders, and systemic ADAM10 concentration may potentially be used as a prognostic biomarker.
- Our study has shown that serum ADAM10 level and its substrate is increased in type 2 diabetes and is associated with glycemia and insulin therapy. This may potentially affect the specificity of systemic ADAM10 level as a biomarker.

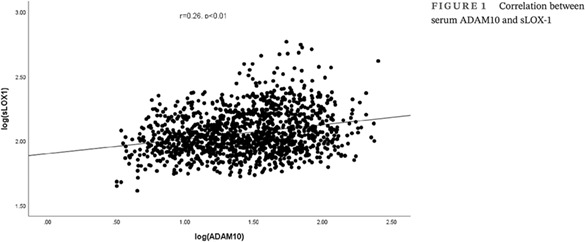

## 摘要

**背景:**

解整合素金属蛋白酶10(ADAM10)是一种1型跨膜蛋白, 负责裂解多种细胞表面分子, 参与阿尔茨海默病, 动脉粥样硬化, 炎症性和肿瘤性疾病的发病。已有研究表明, 全身性ADAM10浓度有可能被用作预后生物标志物。由于高糖在体外可以上调ADAM10的表达, 我们研究了2型糖尿病是否会影响血清ADAM10及其底物‐‐血凝素氧化低密度脂蛋白受体(LOX‐1)的水平。

**方法:**

研究对象为1091例2型糖尿病患者和358例年龄匹配的健康对照组。用双抗体夹心法测定血清ADAM10浓度和解整合素金属蛋白酶裂解LOX‐1释放的可溶性LOX‐1(sLOX‐1)。

**结果:**

糖尿病患者血清ADAM10水平较正常对照组升高[分别为40.5 (22.3‐65.7) ng/ml vs10.3(7.0‐17.9) ng/ml, P<0.01], 以胰岛素治疗组最高。多元线性回归分析显示, 糖化血红蛋白, 年龄, 体重指数和胰岛素用量是ADAM10水平的独立决定因素。糖尿病患者血清ADAM10水平升高的同时伴有血清sLOX‐1的变化。糖尿病患者血清sLOX‐1水平显著高于对照组[分别为110 (89‐153) pg/ml和104(85‐138) pg/ml, P<0.01], 且血清ADAM10水平与sLOX‐1水平显著相关(r=0.26, p<0.01)。

**结论:**

2型糖尿病患者血清ADAM10水平升高, 且与血糖和胰岛素治疗有关, 可能影响ADAM10作为生物标志物的特异性。

## 1 INTRODUCTION

The proteinase family a disintegrin and metalloproteases (ADAM) are type 1 transmembrane proteins and act as sheddases. ADAM can cleave a variety of cell surface molecules and thereby alter the function(s) of their substrates. Ectodomain shedding can either lead to activation or inactivation, and in some instances, the released soluble fragments may also have biological effects.^1^, ^2^ Any dysregulation in the proteolytic activity of ADAM can therefore have pathological consequences.^3^, ^4^ ADAM10 is one of the best studied metalloproteases, and it can cleave almost 100 different substrates.^1^, ^2^, ^3^, ^4^, ^5^ In addition, ADAM10 itself can also be cleaved and released in extracellular vesicles.^6^ Via cleavage of its substrates, ADAM10 is involved in the regulation of cell adhesion, chemotaxis, inflammation, and apoptosis.^1^, ^2^, ^3^, ^4^, ^5^ It has been shown that abnormalities in the proteolytic activity of ADAM10 may play a pathophysiological role in conditions like Alzheimer disease, atherosclerosis, and inflammatory and malignant disorders.^7^, ^8^, ^9^, ^10^, ^11^ On top of its potential as a possible therapeutic target, the circulating level of ADAM10 and/or its substrates may be useful as a diagnostic as well as prognostic biomarker for cardiovascular disease, Alzheimer disease, and cancer.^12^ Petricia et al recently reported that urinary podocyte‐associated ADAM10 might be a biomarker of early diabetic nephropathy.^13^

Since ADAM10 may potentially be used as a biomarker, it is important to determine whether ADAM10 concentration is affected by comorbid conditions. Recent data have suggested that the expression of ADAM10 might be influenced by the diabetic milieu. Incubation of human arterial smooth muscle cells with high glucose induced an increase in ADAM10 mRNA and protein expression in vitro.^14^ In diabetic minipig animal models, ADAM10 expression was found to be increased in coronary artery in‐stent restenosis segments.^14^ Experimental studies have also shown that advanced glycation end products can upregulate the expression of ADAM10.^14^, ^15^ Furthermore, insulin has been shown to stimulate ADAM10 expression and activity.^16^, ^17^ We therefore firstly determined whether type 2 diabetes was associated with changes in serum ADAM10 levels and examined the effect of exogenous insulin therapy on ADAM10 levels. Secondly, we investigated if changes in serum levels of ADAM10 in diabetes were associated with changes in its substrates. Out of the many substrates of ADAM10,^1^, ^2^, ^3^, ^4^, ^5^ we selected to measure the lectin‐like oxidized low‐density lipoprotein receptor 1 (LOX‐1), the main receptor for oxidized LDL uptake in endothelial cells. LOX‐1 has been implicated in endothelial activation and dysfunction in atherosclerosis.^18^, ^19^ Cleavage of LOX‐1 by ADAM releases the soluble form of LOX‐1 (sLOX‐1) which is one of the novel biomarkers of cardiovascular disease.^19^

## 2 METHODS

Type 2 diabetic subjects with and without insulin therapy were recruited from the diabetes clinics at Queen Mary Hospital. All subjects had to have stable glycemic control with no change in antidiabetic therapy for the preceding 3 months. Major exclusion criteria were non‐Chinese descent, type 1 diabetes, malignancy or major illness with limited life expectancy, and any hospitalization in the preceding 3 months. Clinical data were retrieved form medical records, and cardiovascular disease was defined as documented evidence of coronary heart disease (angina, myocardial infarction, significant stenosis on coronary angiography, or coronary revascularization), ischemic stroke, and/or peripheral vascular disease. The diagnosis of retinopathy was based on fundoscopic findings by ophthalmoscopy and peripheral neuropathy by assessment of vibration perception threshold. Healthy nondiabetic control subjects were recruited from the community. The study was approved by the ethics committee of the University of Hong Kong, and it conforms to the provisions of the Declaration of Helsinki in 1995. Informed consent was obtained from all subjects.

Fasting blood samples were taken for the measurement of glucose, HbA1c, lipids, creatinine, ADAM10, and sLOX‐1. Estimated glomerular filtration rate (eGFR) was calculated using the Chronic Kidney Disease Epidemiology Collaboration equation. Serum levels of ADAM10 and sLOX‐1 were measured using commercially available solid‐phase enzyme‐linked immunosorbent assay kits (ELISA) (R&D systems, Minneapolis, Minnesota) according to the manufacturer's protocol. Absorbance reading against the concentration of sLOX‐1 and ADAM10 were quantified within 30 minutes by an ELISA reader at 450 nm. The intra‐assay and inter‐assay coefficients for ADAM10 were 3.8% and 7.3%, respectively, and sLOX‐1 4.6% and 7.7%, respectively.

Results were expressed as mean and standard deviation or as median and interquartile range if the data were not normally distributed. Skewed data were logarithmically transformed before analyses were made. Comparisons between the two groups were done using an independent sample *t* test. Pearson's correlation coefficient and multiple linear regression analysis were used to test the relationships between variables.

## 3 RESULTS

A total of 1091 individuals with type 2 diabetes and 358 healthy control subjects were recruited. The clinical characteristics are shown in Table 1. Serum ADAM10 levels were significantly higher in individuals with diabetes than in the control and remained significant even after adjusting for body mass index (BMI) (*P* < .01). There were no gender differences in serum ADAM10 levels. Since peroxisome proliferator‐activated receptor (PPAR) alpha agonists are known to activate ADAM10,^20^ excluding those subjects on fibrate from the analysis did not change the results either. The mean HbA1c was 8.5% ± 1.6 in subjects with diabetes, and glycemic control was suboptimal. We therefore compared serum ADAM10 levels in subjects with HbA1c ≤ 7.5% (n = 296) with the control, and serum ADAM10 remained significantly increased (41.0 ng/mL [21.8‐68.9] vs 10.3 ng/mL [7.0‐17.9], respectively; *P* < .01). To determine whether serum ADAM10 levels were associated with diabetic complications, individuals with cardiovascular disease (n = 112) were compared to those without (n = 979). Serum ADAM10 level was higher in subjects with cardiovascular disease (51.3 ng/mL [27.2‐74.1] vs 40.7 ng/mL [22.9‐64.7], respectively; *P* = .03). There were no significant differences in serum ADAM10 levels between those with and without microvascular complications (data not shown).

**TABLE 1:** Clinical characteristics, serum ADAM10, and sLOX‐1 in control and subjects with type 2 diabetes

	Control	T2DM
n	358	1091
Age (y)	53.4 ± 5.6	54.3 ± 8.7
M/F (%)	49.5/50.5	51.1/48.9
Duration of diabetes (y)	–	13 ± 7
BMI (kg/m^2^)	24.3 ± 4.0	25.9 ± 4.0*
Smoker (%)	9.9	11.5
Hypertension (%)	–	67.2
Cardiovascular disease (%)	–	10.3
Retinopathy (%)	–	27.9
Neuropathy (%)	–	16.5
Chronic kidney disease ≥ stage 3 (%)	–	23.0
Oral antidiabetic agents (%)	–	80.6
Insulin (%)	–	37.7
Statin (%)	–	25.9
Fibrate (%)	–	5.8
Systolic BP (mm Hg)	121 ± 18	132 ± 20*
Diastolic BP (mm Hg)	76 ± 10	77 ± 9
Fasting glucose (mmol/L)	5.1 ± 0.6	8.6 ± 2.8*
HbA1c (%)	5.7 ± 0.5	8.5 ± 1.5*
eGFR (mL/min/1.73 m^2^)	77.0 (69.0‐86.4)	72.4 (60.2‐85.4)
Total cholesterol (mmol/L)	5.28 ± 0.84	4.93 ± 1.00*
Triglyceride (mmol/L)	0.90 (0.70‐1.40)	1.30 (0.90‐1.90)*
LDL‐C (mmol/L)	3.32 ± 0.78	2.97 ± 0.92*
HDL‐C (mmol/L)	1.40 ± 0.37	1.23 ± 0.34*
ADAM10 (ng/mL)	10.3 (7.0‐17.9)	40.5 (22.3‐65.7)*
sLOX‐1 (pg/mL)	104 (85‐138)	110 (89‐153)*

*Note*: Data are expressed as mean ± SD or median (interquartile range).

Abbreviations: ADAM10, a disintegrin and metalloprotease 10; BMI, body mass index; BP, blood pressure; eGFR, estimated glomerular filtration rate; HbA1c, glycosylated hemoglobin; HDL‐C, high‐density lipoprotein cholesterol; LDL‐C, low‐density lipoprotein cholesterol; sLOX‐1, soluble form of lectin‐like oxidized low‐density lipoprotein receptor; T2DM, type 2 diabetes.

*P < .01 vs control.

As insulin stimulates ADAM10 expression and activity,^16^, ^17^ we investigated whether exogenous insulin therapy had any effect on serum ADAM10 levels. Individuals with diabetes receiving insulin therapy were matched with subjects receiving oral antidiabetic agents for age and gender. Serum ADAM10 levels were increased in insulin‐treated subjects compared to those on oral antidiabetic agents (Table 2). These differences remained significant after adjusting for duration of diabetes and HbA1c (*P* < .05). There was a significant correlation (*r* = 0.38, *P* < .01) between serum ADAM10 levels and total daily insulin dose.

**TABLE 2:** Clinical characteristics, serum ADAM10, and sLOX‐1 in type 2 diabetes with and without insulin therapy

	T2DM not on insulin	T2DM on insulin
n	400	411
Age (y)	55.8 ± 8.8	55.8 ± 8.5
M/F (%)	51.5/48.5	52.1/47.9
Duration of diabetes (y)	13 ± 5	15 ± 7*
BMI (kg/m^2^)	25.7 ± 3.8	25.9 ± 4.0
Smoker (%)	10.4	13.6
Hypertension (%)	64.4	72.5*
Cardiovascular disease (%)	8.0	12.7
Retinopathy (%)	25.9	39.7*
Neuropathy (%)	13.0	19.7
Chronic kidney disease ≥ stage 3 (%)	18.3	30.0*
Oral antidiabetic agents		
Metformin (%)	88.3	70.7*
Sulfonylureas (%)	89.5	4.6**
Acarbose (%)	6.3	0.1
Thiazolidinediones (%)	0.2	0.1
Dipeptidyl peptidase IV inhibitors (%)	10.3	4.0
Sodium glucose cotransporter 2 inhibitors (%)	2.1	0.9
Total daily insulin dose (units)	‐	38 ± 17
Statin (%)	18.8	24.7
Fibrate (%)	5.3	5.4
Systolic BP (mm Hg)	129 ± 20	131 ± 22
Diastolic BP (mm Hg)	78 ± 9	75 ± 10
Fasting glucose (mmol/L)	8.5 ± 2.4	8.7 ± 3.2
HbA1c (%)	8.2 ± 1.4	9.1 ± 1.7**
eGFR (mL/min/1.73m^2^)	73.6 (62.3‐85.2)	70.1 (54.5‐86.2)**
Urine albumin to creatinine ratio (mg/mmol)	1.9 (1.3‐4.8)	2.6 (1.6‐10.0)**
Total cholesterol (mmol/L)	4.99 ± 0.95	4.79 ± 0.98*
Triglyceride (mmol/L)	1.30 (0.90‐2.00)	1.30 (0.90‐1.90)
LDL‐C (mmol/L)	3.02 ± 0.87	2.83 ± 0.90*
HDL‐C (mmol/L)	1.23 ± 0.31	1.25 ± 0.37
ADAM10 (ng/mL)	40.9 (21.9‐60.4)	46.5 (25.8‐68.6)*
sLOX‐1 (pg/mL)	109 (88‐141)	119 (92‐165)**

*Note:* Data are expressed as mean ± SD or median (interquartile range).

Abbreviations: ADAM10, a disintegrin and metalloprotease 10; BMI, body mass index; BP, blood pressure; eGFR, estimated glomerular filtration rate; HbA1c, glycosylated hemoglobin; HDL‐C, high‐density lipoprotein cholesterol; LDL‐C, low‐density lipoprotein cholesterol; sLOX‐1, soluble form of lectin‐like oxidized low‐density lipoprotein receptor; T2DM, type 2 diabetes.

*P < .05; **P < .01 vs T2DM not on insulin.

Further analysis was performed to evaluate which clinical parameters influenced serum ADAM10 levels. In all subjects, serum ADAM10 level correlated with age (*r* = 0.28, *P* < .01), BMI (*r* = 0.14, *P* < .01), HbA1c (*r* = 0.45, *P* < .01), fasting glucose (*r* = 0.38, *P* < .01), high‐density lipoprotein cholesterol (HDL‐C) (*r* = −0.10, *P* < .01), and log(eGFR) (*r* = −0.11, *P* < .01). Repeating the analysis in the diabetic cohort alone showed that serum ADAM10 level also correlated with age (*r* = 0.11, *P* < .01), HbA1c (*r* = 0.21, *P* < .01), fasting glucose (*r* = 0.13, *P* < .01), and log(eGFR) (*r* = −0.08, *P* < .05). The associations with BMI and HDL‐C were no longer significant, and there was no correlation with urine albumin to creatinine ratio. On multiple linear regression analysis of all subjects together, only HbA1c, age, BMI, and insulin use were independent determinants of ADAM10 level (Table 3).

**TABLE 3:** Determinants of serum ADAM10 on multiple linear regression analysis

	Regression coefficient	SE of regression coefficient	*P* value
Age	0.007	0.001	<.001
Gender	0.012	0.019	.54
BMI	0.005	0.003	.03
Smoking (No/Yes)	−0.007	0.005	.13
HbA1c	0.068	0.006	<.001
HDL‐C	−0.054	0.028	.06
Log (eGFR)	0.129	0.070	.06
Insulin (No/Yes)	0.096	0.024	<.001

Abbreviations: ADAM10, a disintegrin and metalloprotease 10; BMI, body mass index; eGFR, estimated glomerular filtration rate; HbA1c, glycosylated hemoglobin; HDL‐C, high‐density lipoprotein cholesterol.

**FIGURE 1:**
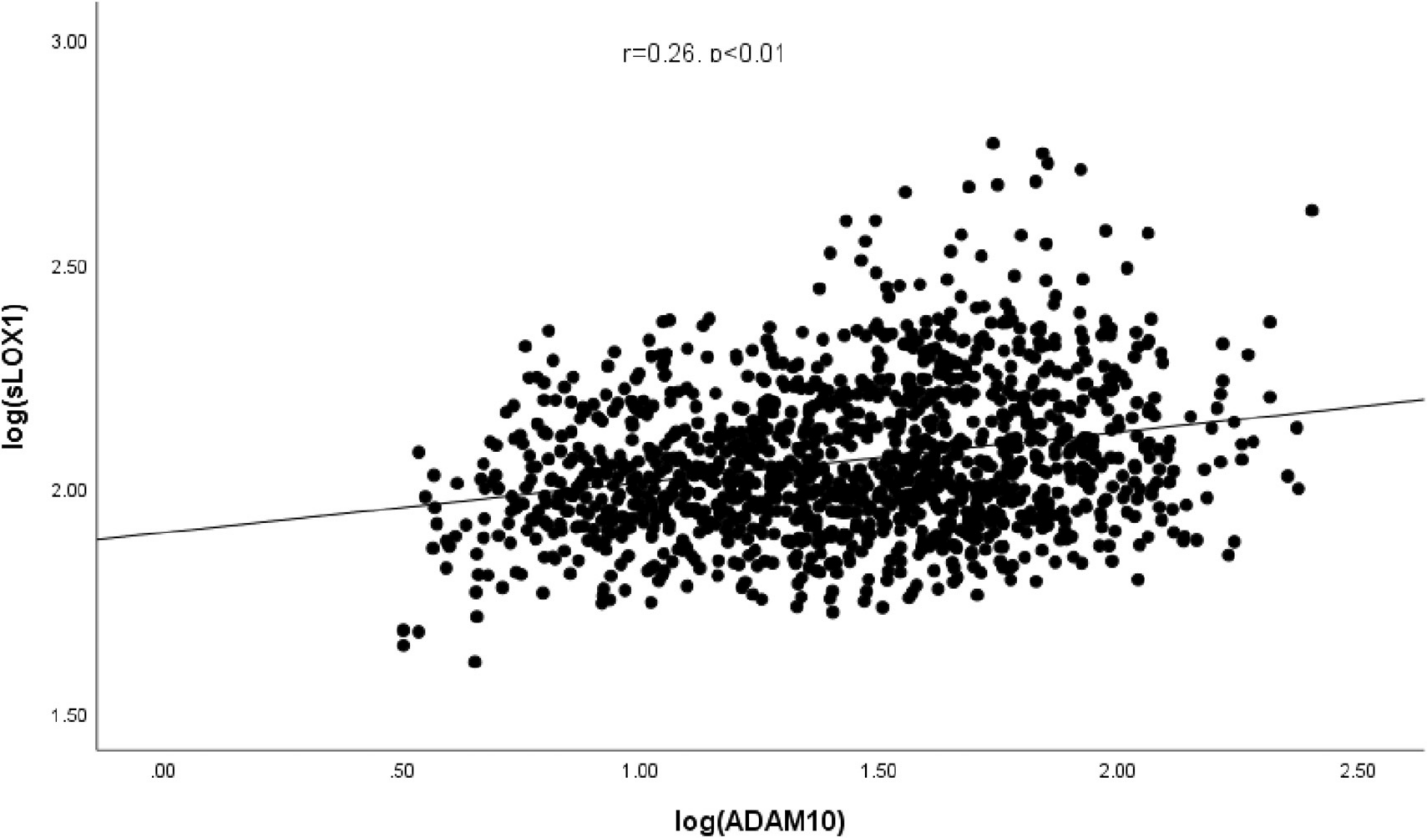
Correlation between serum ADAM10 and sLOX‐1

We proceeded to evaluate whether changes in serum ADAM10 levels were associated with sLOX‐1 concentration. Subjects with diabetes had higher serum sLOX‐1 than the control (Table 1). Moreover, in subjects with diabetes, those with cardiovascular disease (n = 112) had higher serum sLOX‐1 than those without cardiovascular disease (n = 979) (120 pg/mL [90‐173] vs 111 pg/mL [89‐154], respectively; *P* = .04). In all subjects, there was a significant correlation between serum ADAM10 and sLOX‐1 (*r* = 0.26, *P* < .01) (Figure 1).

## 4 DISCUSSION

ADAM10 is a multifunctional protease and has been evaluated as a potential therapeutic target and biomarker because of its involvement in the pathogenesis of Alzheimer disease, atherosclerosis, and inflammatory and neoplastic disorders.^7^, ^8^, ^9^, ^10^, ^11^ The effects of ADAM10 activity can differ between pathological conditions and can be either favorable or harmful. The expression and activity of ADAM10 is regulated at the transcriptional, translational, and posttranscriptional level.^21^ Improving our understanding on the regulation of ADAM10 is therefore vital and a crucial step toward evaluating its value in therapeutic contexts. Our study is the first study investigating systemic levels of ADAM10 in people with type 2 diabetes. We have shown that serum ADAM10 level is increased in these subjects. Consistent with experimental studies showing that both glucose and advanced glycation end products can upregulate ADAM10 expression,^14^, ^15^ we found a significant association between serum levels of ADAM10 and HbA1c as well as fasting glucose. In addition, total daily insulin dosage was associated with systemic concentration of ADAM10, and insulin‐treated individuals had higher ADAM10 concentrations than those on oral‐antidiabetic agents. This is in keeping with in vitro studies showing that insulin stimulates ADAM10 expression and activity.^17^ A recent study has reported that tissue expression of ADAM10 is increased in diabetes. Herman‐Edelstein et al demonstrated that ADAM10 expression was upregulated in the right atrial appendage biopsies collected from diabetic subjects undergoing coronary artery bypass graft surgery.^22^ Whether systemic ADAM10 concentration reflects tissue ADAM10 expression warrants further investigation.

Moreover, we have shown that the observed increase in systemic ADAM10 level in type 2 diabetes was accompanied by changes in its substrate concentration, and serum ADAM10 level correlated with sLOX‐1 in our study. LOX‐1 is one of the substrates of ADAM10 and plays a pro‐atherogenic role in the development and progression of cardiovascular disease.^23^ Since ADAM10 can only interact with its substrate if it is expressed simultaneously and within the same cellular location as its substrate, the ultimate consequences of ADAM10 activation is determined by the patterns of tissue expression and time courses of ADAM10 and its substrates. ADAM10 is ubiquitously expressed, and in the blood vessels, ADAM10 is found in endothelial cells, leukocytes, and vascular smooth muscle cells.^8^ The normal human vessel wall has low levels of ADAM10, but these increase markedly during plaque development and progression. In advanced atherosclerotic lesions, ADAM10 is highly expressed in plaque microvessels and macrophages/foam cells.^24^ Likewise, LOX‐1 expression is minimal under physiological conditions. In early atherosclerotic lesions, LOX‐1 is mainly expressed in endothelial cells, and the expression of LOX‐1 increases and extends to smooth muscle cells and macrophages in more advanced lesions. Hence, the temporospatial expression of ADAM10 parallels that of LOX‐1 in the vasculature. The correlation between ADAM10 and sLOX‐1 is not very strong in our study, probably because LOX‐1 is also cleaved by ADAM17 and matrix metalloproteinases. Proteolysis is one of the main mechanisms controlling the function of LOX‐1, and it has been proposed that the development of atherosclerotic plaques may be reduced by stimulating LOX‐1 proteolysis. The decrease in cell surface LOX‐1 through enhanced cleavage by ADAM and matrix metalloproteinases will lessen the uptake of oxidized LDL. Moreover, the pro‐atherogenic signaling of LOX‐1 will also be diminished.^18^

Manipulating ADAM10 as a potential therapeutic target is fraught with difficulties because ADAM10 is ubiquitously expressed. On the other hand, circulating levels of ADAM10 and/or its substrates may be a useful diagnostic as well as prognostic biomarker for a number of conditions.^12^ For instance, Feng et al have shown that plasma ADAM10 level is a useful prognostic biomarker of intracerebral hemorrhage.^25^ ADAM10 might also be a potential blood biomarker for Alzheimer disease because it is the major alpha‐secretase that cleaves amyloid precursor protein in neurons. Changes in plasma levels of ADAM10 have been shown to predict the deterioration of cognitive function in older adults.^26^ Serum ADAM10 levels have also been evaluated as a biomarker for malignancy like colorectal cancer.^27^ In diabetes, it has been suggested that urinary ADAM10 might be a biomarker of early diabetic nephropathy.^13^ We have demonstrated that serum ADAM10 is increased in type 2 diabetes, but our study does not have sufficient power to investigate the relationship between diabetic vascular complications and ADAM10. However, our data have demonstrated that caution needs to be exercised if systemic ADAM10 level is being considered as a potential biomarker for other conditions like Alzheimer disease in individuals with diabetes. Circulating levels of ADAM10 can be affected by glycemia and by treatment modalities like exogenous insulin therapy. Hence, the ubiquitous nature of ADAM10 expression and the upregulation in disease states may affect its use as a biomarker in situations with co‐existing comorbidities like diabetes.

Our study has several limitations. It is cross‐sectional in design, and we can only demonstrate associations and not causal relationships. We have studied only one member of the ADAM family, and it is not known whether systemic ADAM10 concentration reflects tissue ADAM10 expression. Furthermore, since ADAM10 is ubiquitously expressed, which tissue(s) are the major source of systemic ADAM10 is unclear. The precise in vivo and particularly cell‐specific and tissue‐specific effects of ADAM10 in diabetes still largely need to be determined, and our study is not powered to evaluate the role of ADAM10 as a marker of diabetic vascular complications. Our cohort of subjects with diabetes was recruited from a secondary/tertiary referral center, and they were likely to be more complex than those individuals treated in primary care or in the general population. Our results may not be generalized to other patient populations.

In conclusion, serum concentration of ADAM10 is increased in type 2 diabetes and is associated with glycemia and insulin therapy. This may potentially affect the specificity of systemic ADAM10 level as a biomarker when diabetes is present as a comorbid condition.

## CONFLICT OF INTEREST

No conflict of interest to declare.
